# Acute Liver Toxicity Modifies Protein Expression of Glutamate Transporters in Liver and Cerebellar Tissue

**DOI:** 10.3389/fnins.2020.613225

**Published:** 2021-01-06

**Authors:** Catya Jiménez-Torres, Hoda El-Kehdy, Luisa C. Hernández-Kelly, Etienne Sokal, Arturo Ortega, Mustapha Najimi

**Affiliations:** ^1^Laboratorio de Neurotoxicología, Centro de Investigación y de Estudios Avanzados del Instituto Politécnico Nacional (Cinvestav-IPN), Departamento de Toxicología, Mexico City, Mexico; ^2^Laboratory of Pediatric Hepatology and Cell Therapy, UCLouvain, Institut de Recherche Expérimentale et Clinique (IREC), Brussels, Belgium

**Keywords:** GLAST/EAAT1, GLT1/EAAT2, glutamine synthetase, glial cell, liver injury, carbon tetrachloride

## Abstract

Glutamate is the main excitatory amino acid acting at the level of pre and postsynaptic neurons, as well as in glial cells. It is involved in the coordinated modulation of energy metabolism, glutamine synthesis, and ammonia detoxification. The relationship between the functional status of liver and brain has been known for many years. The most widely recognized aspect of this relation is the brain dysfunction caused by acute liver injury that manifests a wide spectrum of neurologic and psychiatric abnormalities. Inflammation, circulating neurotoxins, and impaired neurotransmission have been reported in this pathophysiology. In the present contribution, we report the effect of a hepatotoxic compound like CCl_4_ on the expression of key proteins involved in glutamate uptake and metabolism as glutamate transporters and glutamine synthetase in mice liver, brain, and cerebellum. Our findings highlight a differential expression pattern of glutamate transporters in cerebellum. A significant Purkinje cells loss, in parallel to an up-regulation of glutamine synthetase, and astrogliosis in the brain have also been noticed. In the intoxicated liver, glutamate transporter 1 expression is up-regulated, in contrast to glutamine synthetase which is reduced in a time-dependent manner. Taken together our results demonstrate that the exposure to an acute CCl_4_ insult, leads to the disruption of glutamate transporters expression in the liver-brain axis and therefore a severe alteration in glutamate-mediated neurotransmission might be present in the central nervous system.

## Introduction

The liver is essential for the balance of metabolism in the body. It is responsible for protein, carbohydrate and lipid metabolism, glucose homeostasis, bile secretion, and detoxification. This organ provides energy to the whole body by managing the systemic supply of nutrients. It is also recognized as a mediator of systemic and local innate immunity as well as an important site of immune regulation (Nemeth et al., 2009; Gu and Manautou, 2012). Acute liver injury (ALI) is characterized by a systemic elevation of transaminases levels related to hepatocellular damage, and is associated with impaired liver functions in patients without previous liver diseases. ALI is commonly related to drug hepatotoxicity, however, it is applied to other clinical presentations and it is considered the first stage in the clinical course of acute liver failure that eventually develops in Hepatic Encephalopathy (HE) (Wendon et al., 2017). The relationship between the functional status of liver and brain has been reported for many years. The most widely recognized aspect of this relationship is the brain dysfunction caused by liver insufficiency and manifested *via* a wide spectrum of neurologic and psychiatric abnormalities ranging from subclinical alterations to coma. Although, it is potentially reversible, it may result in a significantly increased morbidity, with an impact on liver transplantation outcome and mortality (Lewis and Howdle, 2003). The pathogenesis of HE is poorly understood and several factors such as systemic and local inflammation, circulating neurotoxins (manganese and ammonia, neurosteroids), lactate accumulation, changes in brain energy metabolism, alterations of the blood-brain barrier and impaired neurotransmission have been shown to play important roles (Mans et al., 1994; Krieger et al., 1995; Zwingmann et al., 2003; Shawcross et al., 2007; Ahboucha and Butterworth, 2008; Ferenci, 2017).

Glutamate (Glu) is the main excitatory neurotransmitter whose function requires the involvement of both neurons and glial cells (Martínez-Lozada and Ortega, 2015). Glu concentrated in vesicles in the presynaptic terminal by the vesicular glutamate transporters, is released into the synaptic cleft upon depolarization of the presynaptic membrane and activates its metabotropic and ionotropic (NMDA, AMPA, and Kainate) receptors both at the pre and postsynaptic membranes, a process significantly crucial for synaptic plasticity. Glu-induced depolarization leads to a postsynaptic excitatory potential which facilitates the generation of an action potential (Kew and Kemp, 2005). Since the excess of Glu leads to hyper-activation of its receptors, a phenomenon known as excitotoxicity, the levels of Glu in the synaptic clef are tightly regulated by the sodium-dependent glutamate transporters, members of the solute carrier family 1 (SLC1), also named Excitatory Amino Acid Transporters (EAATs), which are highly expressed in glial cells. Glu uptake by these cells maintains the extracellular Glu concentration in the low micromolar range (Danbolt, 2001). Once internalized into astrocytes, Glu is amidated by Glutamine Synthetase (GS) to glutamine (Gln), which is then released, taken up by neurons and converted back to Glu and used once again for neurotransmission; such mechanism is known as the Glu-Gln shuttle (McKenna et al., 2012; Martínez-Lozada and Ortega, 2015).

The disruption of the activity and expression of GS and the two main Glu transporters in the Central Nervous System (CNS), the Na^+^- dependent Glu and aspartate transporter (GLAST/EAAT1) and Glu transporter1 (GLT-1/EAAT2) have been described in advanced liver injuries such as HE and hyperammonemia (Waniewski, 1992; Butterworth, 2002; Bémeur and Butterworth, 2013; Hu et al., 2018; Sohn et al., 2018). However, the effects on Glu transporters expression at early stages of liver injury are poorly characterized. Carbon tetrachloride (CCl_4_) is commonly used to induce acute and chronic liver damage *in vivo*. A single dose of CCl_4_ induces hepatomegaly, fat deposition, increased levels of serum urea and liver enzymatic activities, as well as histopathological liver damage with unicellular necrosis (Korsrud et al., 1972), while long-term exposure causes marked hepatotoxicity with fibrosis, bile duct overgrowth, cirrhosis, and even hepatocellular carcinoma (Scholten et al., 2015). Since the mechanism of action of CCl_4_ and its metabolites promote lipid peroxidation, mitochondrial dysfunction, oxidative and nitrosative stress, inflammation by activation of Tumor Necrosis Factor α, Interleukin (IL)−1, IL-6, IL-10 and the release of Nitric Oxide (NO) (Weber et al., 2003), features that are closely involved in the pathophysiology of ALI, we used this experimental model to characterize a plausible effect on Glu transporters and metabolism in liver, brain and cerebellum. Our results propose the disruption of Glu turnover in cerebellum and brain and further support the notion of a brain-liver axis.

## Materials and Methods

### Materials

Carbon tetrachloride reagent grade 99.9% (319,961) was obtained from Sigma- Aldrich. BCA Protein Assay kit (23,227) was provided from Pierce Thermo Fisher Scientific. SDS-PAGE reagents were obtained from Bio-Rad. The reagents used for IHC, Avidin/Biotin Blocking Kit (SP-2001) and M.O.M. elite peroxidase kit (BMK-2202) were purchased from Vector Laboratories.

### Animals Treatment

**Figure 1:**
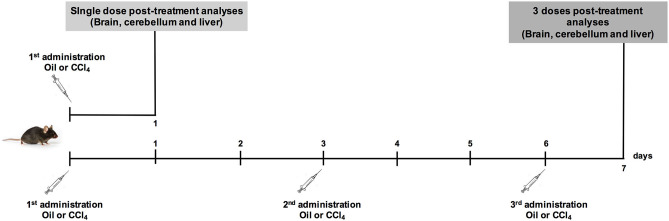
The Experimental model of CCl_4_ -induced acute liver injury that was applied in the current study.

Experiments performed in this study were approved by the local ethical review board (2015/UCL/MD/12). C57BL/6J wild type adult male mice (8 weeks of age) were subjected to a 12 h day-night rhythm with free access to food and water. Acute liver injury was induced with an intraperitoneal injection of CCl_4_. The treatments were divided in one dose or three doses of CCl_4_ (Figure 1). Three to four control and five CCl_4_ treated mice were used per group for these experiments. Animals were randomly assigned to each group and administrated with an intraperitoneal injection of 100 μL of CCl_4_ dissolved in corn oil as vehicle (10%, v/v, 5 mL/kg) per 20 g of body weight. The control mice were administrated with corn oil. Animals were sacrificed 1 and 7 days later. Livers and brains (forebrain and cerebellum) were recovered for analyses. The analysis of the brain tissue was performed separately, where the forebrain corresponds to brain sample.

### Histology and Immunohistochemistry

Specimens were fixed in formalin for 24 h and embedded in paraffin. Liver sections (5 μm-thick sections) were deparaffinized and stained with hematoxylin and eosin (H&E) or Picrosirius red to evaluate histopathological damage and collagen deposition. Immunohistochemistry was performed on 5 μm-thick liver sections that were deparaffinized and rehydrated in a graded alcohol series. After endogenous peroxidase activity, blockade by incubation for 15 min in a 3% hydrogen peroxide methanol solution, slices were incubated with citric acid monohydrate solution (pH 6.0) at 97°C for 15 min for antigen retrieval. Non-specific immuno-staining was prevented by incubation in PBS buffer containing 1% normal goat serum for 1 h at room temperature. Slices were thereafter incubated with polyclonal Anti-GS antibody at a dilution of 1:1000 at 37°C for 1 h (Table 1). Staining was visualized by EnVision + System-HRP Labeled Polymer Anti-Rabbit (DAKO) using diamino- benzidine (Sigma, Belgium) as a chromogenic substrate. The nuclei were counterstained using Mayer's hematoxylin for 1 min. Preparations were then mounted for microscopic analysis.

**Table 1:** Antibodies used for western blot and immunohistochemistry analyses of mice liver, brain, and cerebellum tissue.

**Antibody**	**Supplier**	**Concentration used**	**Reference**
EAAT1/GLAST	Abcam	1/1000	Ab416
EAAT2/GLT-1	Alomone	1/1000	AGC-022
GS	BD Transduction	1/1000; 1/100	610517
GFAP	Dako	1/1000	Z0334
α-SMA	Abcam	1/250	Ab8211
GAPDH	Sigma	1/5000	G8795
Rabbit	Biotium	1/5000	CF®680
Mouse	Biotium	1/5000	CF®770
Control antigen	Alomone	1 μg/1 μg antibody	AGC-022

### SDS-PAGE and Western Blot

Protein extraction was performed according to the protocol of Gregor and collaborators (Gregor et al., 1988) with minor modifications. The tissue was homogenized in 10 vol of ice-cold 50 mM Tris-acetate pH 7.0 buffer containing 5 mM EDTA and 1 mM PMSF. The suspension was centrifuged for 10 min at 3,000 rpm and 4°C, the first pellet (cytoplasmic fraction) was homogenized and the supernatant was recovered and centrifuged at 14,000 rpm for 20 min at 4°C. The second pellet (membranous fraction) was re-suspended mechanically and sonicated for 55 s at 90%. An aliquot of each fraction was used for protein quantification. The samples were denatured in Laemmli's sample buffer, and an equal amount of proteins (25 μg) was resolved through a 10% SDS-PAGE and then electroblotted onto PVDF membranes. The cytoplasmic fraction was load to detect GS, α-SMA and GFAP protein, while the membranous fraction was load to detect EAATs expression. Blots were stained with Ponceau Red S to check the quality of the transfer and to confirm that protein content was equal in all lanes. Membranes were soaked in PBS to remove the Ponceau Red S and incubated in TBS containing 5% BSA and 0.1% Tween-20 for 60 min to block the excess of non-specific protein binding sites. Membranes were then incubated overnight at 4°C with the primary antibodies (Table 1). Incubation with the primary antibody plus the blocking peptide for Anti-EAAT2 (Table 1) was used as a negative control. After washing three times for 10 min, the blots were incubated with secondary antibodies for 2 h at room temperature. Immunoreactive polypeptides were detected by a near-infrared Odyssey® Imaging System. Densitometry analyses were performed, and data analyzed with Prism, GraphPad Software (San Diego, CA, USA). All blots were normalized by total protein (Coomassie) and GAPDH.

### Neuronal Cell Density Analysis

For quantification of neuronal cell density in the Purkinje layer of cerebellum, hematoxylin-eosin stained sagittal sections were used. For density analysis at least five random fields for each case were quantified. Purkinje neurons were counted using ImageJ software and results were expressed in percentage related to the mean of the vehicle-treated mice (control), considered as 100%.

### Statistical Analyses

Data are expressed as the mean ± standard deviation (SD). An unpaired *t*-test with Welch's correction was performed to determine significant differences between conditions (at the 0.05 level) and a one-way analysis of variance was performed to determine significant differences between conditions. When this analysis indicated significance (at the 0.05 level), Dunnett's multiple comparison analysis was used to determine which conditions were significantly different from the control with the Prism 5, GraphPad Software (San Diego, CA, USA).

## Results

### Validation of Acute Liver Injury Model by CCl_4_ Treatment

**Figure 2:**
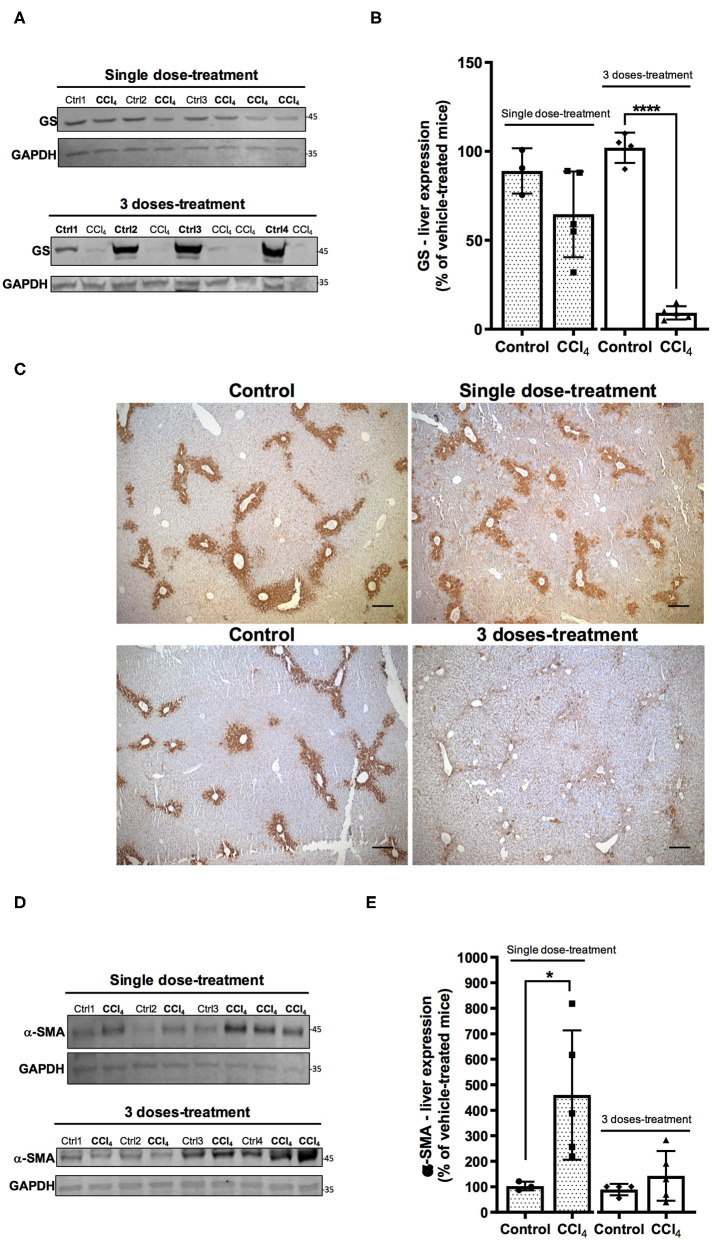
Acute liver injury by CCl_4_ promotes alterations in GS **(A–C)** and α- SMA **(D,E)** protein expression in the mouse liver. **(A)** GS immunoreactivity in the group treated with one dose of CCl_4_ or three doses of CCl_4_. **(B)** quantification of GS protein expression signal intensity. **(C)** GS protein expression was visualized on liver slices by immunohistochemistry using the same antibody as in western blot analyses. Representative images were shown for each mice group (scale: 50 μm). **(D)** α-SMA immunoreactivity in the group treated with one dose or three doses of CCl_4._ **(E)** quantification of α-SMA protein expression signal intensity. Values are the mean ± SD (*n* = 3–5 animals per group). An unpaired *t*-test with Welch's correction was performed to compare between the groups, ****p < 0.0001, *p < 0.05 vs. vehicle-treated mice (control).

CCL_4_ is a toxic chemical widely used to induce liver injury in several laboratory animal models and mimics human hepatotoxicity. The Non-Observable Adverse Effects Levels (NOAEL) calculated in a mouse model after CCl_4_ exposure in corn oil is 1.2 mg/kg of body weight (5 days/week) (WHO, 1997). In our study, mice were exposed to a single or three doses of 5 mg/kg CCl_4_ of body weight (Figure 1). The histological data confirmed through hematoxylin-eosin staining, the hepatic injury 1 day after a single dose of CCl_4_. A normal parenchymal architecture in the vehicle-treated mice was detected, however, 1 day after a single dose of CCl_4_ degeneration of hepatocytes in the centrilobular zone, necrosis and inflammatory foci were observed (Supplementary Figure 1A). After three doses of CCl_4_, the injury around the perivenous zone including necrosis and inflammatory foci was maintained (Supplementary Figure 1A). As expected, collagen deposition was much more evident after three CCl_4_ doses around the perivenous hepatocytes (Supplementary Figure 1B). GS expression as assayed by western blot showed a tendency to a decrease (65 ± 23.87%) in the livers treated with a single dose of CCl_4_ (Figures 2A,B), compared to vehicle-treated mice. Such protein expression was almost abolished (90%) after three doses of CCl_4_ (*p* < 0.0001) (Figures 2A,B). These results are in line with those obtained by immunohistochemistry (Figure 2C). The expression of α-SMA, which is enhanced after trans-differentiation of hepatic stellate cells to myofibroblasts (Kanta et al., 2016) was significantly up-regulated after a single CCl_4_ dose (Figures 2D,E), with four-fold increase in the protein levels (287 ± 88.83%; *p* = 0.034). However, no differences were detected in the α-SMA expression after three doses of CCl_4_ compared to vehicle-treated mice (Figures 2D,E).

### Neuronal Loss and Glial Activation Are Associated to the Acute Liver Toxicity

**Figure 3:**
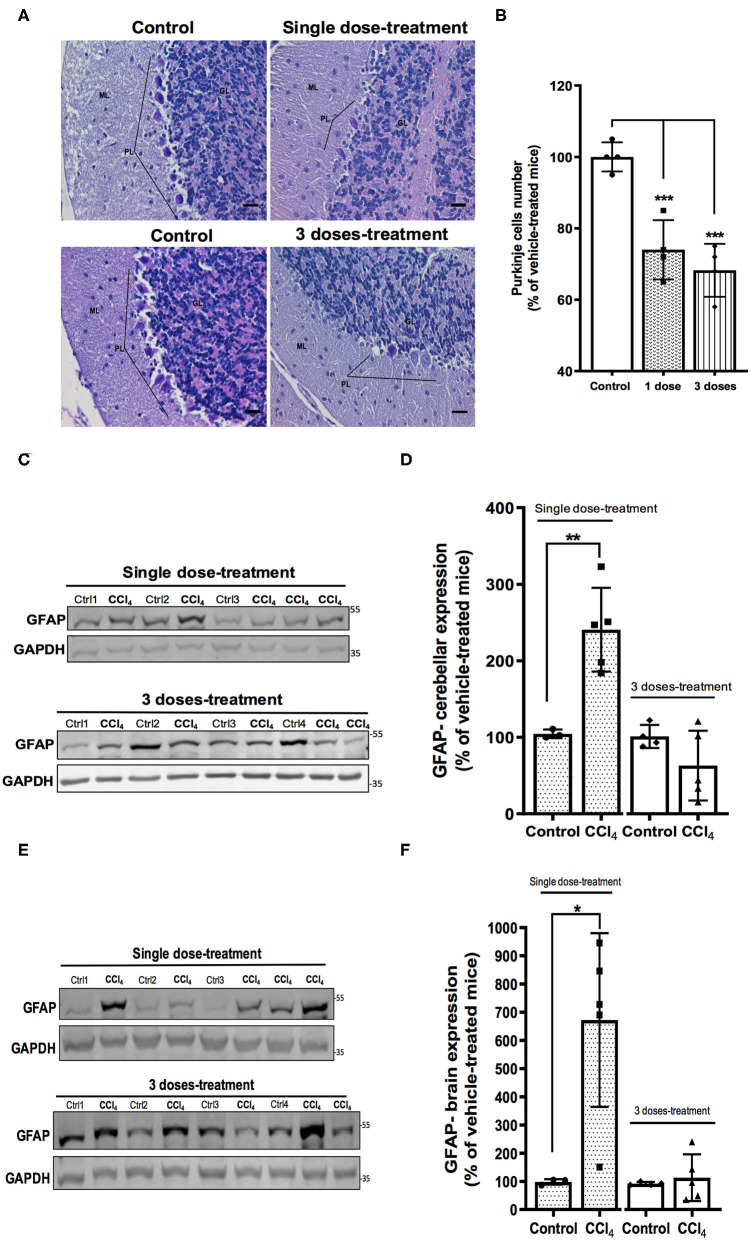
Mice with acute hepatotoxicity show cell loss in the Purkinje layer **(A,B)** and glial activation in cerebellum **(C,D)** and brain **(E,F)**. **(A)** High magnification (400×) representative images of Purkinje layer for each group were presented (scale: 50 μm). **(B)** Quantification of the Neuronal Purkinje cells number. **(C)** GFAP immunoreactivity in cerebellum from mice group treated with one dose of CCl_4_ or three doses of CCl_4_. **(D)** quantification of GFAP protein expression signal intensity in cerebellum tissue. **(E)** GFAP immunoreactivity in brain from mice group treated with one dose of CCl_4_ or three doses of CCl_4_. **(F)** Quantification of GFAP protein expression signal intensity in brain tissue. Results are expressed as mean ± SD (*n* = 3–5 animals per group). One-way ANOVA with Dunnett's *post hoc* test was performed to compare all groups in the cell loss. An unpaired *t*-test with Welch's correction was performed to compared between groups, ***p < 0.0005, **p < 0.01, *p < 0.05 vs. vehicle-treated mice (control).

It has been reported at the early stages of liver damage as steatohepatitis, loss of the cerebellar neurons (Balzano et al., 2018). Therefore, we evaluated the histological state of the cerebellar molecular layer after CCl_4_ treatments. Hematoxylin-eosin staining shows neuronal loss in Purkinje layer in the cerebellum of mice treated with CCl_4_. The number of Purkinje neurons was reduced by 26% (74 ± 8.02%) and 32% (68 ± 7.41%) after single dose and three doses of CCl_4_, respectively, as compared to vehicle-treated mice (100 ± 4.08%) (*p* = 0.0002) (Figures 3A,B). Glial activation was also assessed in brain and cerebellum tissues. Immunoblotting was performed using the astroglial marker GFAP. Mice treated with a single dose of CCl_4_ had a significant increase in GFAP protein expression in both cerebellum (209 ± 33.08%; *p* = 0.0048) (Figures 3C,D) and brain (575 ± 301.90; *p* = 0.014) (Figures 3E,F) as compared to vehicle-treated mice. In contrast, no significant changes were detected in GFAP protein levels after three doses with CCl_4_, neither in cerebellum or brain (Figures 3C–F).

### Glutamate Transporters Expression Is Impaired in CNS After Acute Hepatotoxicity

**Figure 4:**
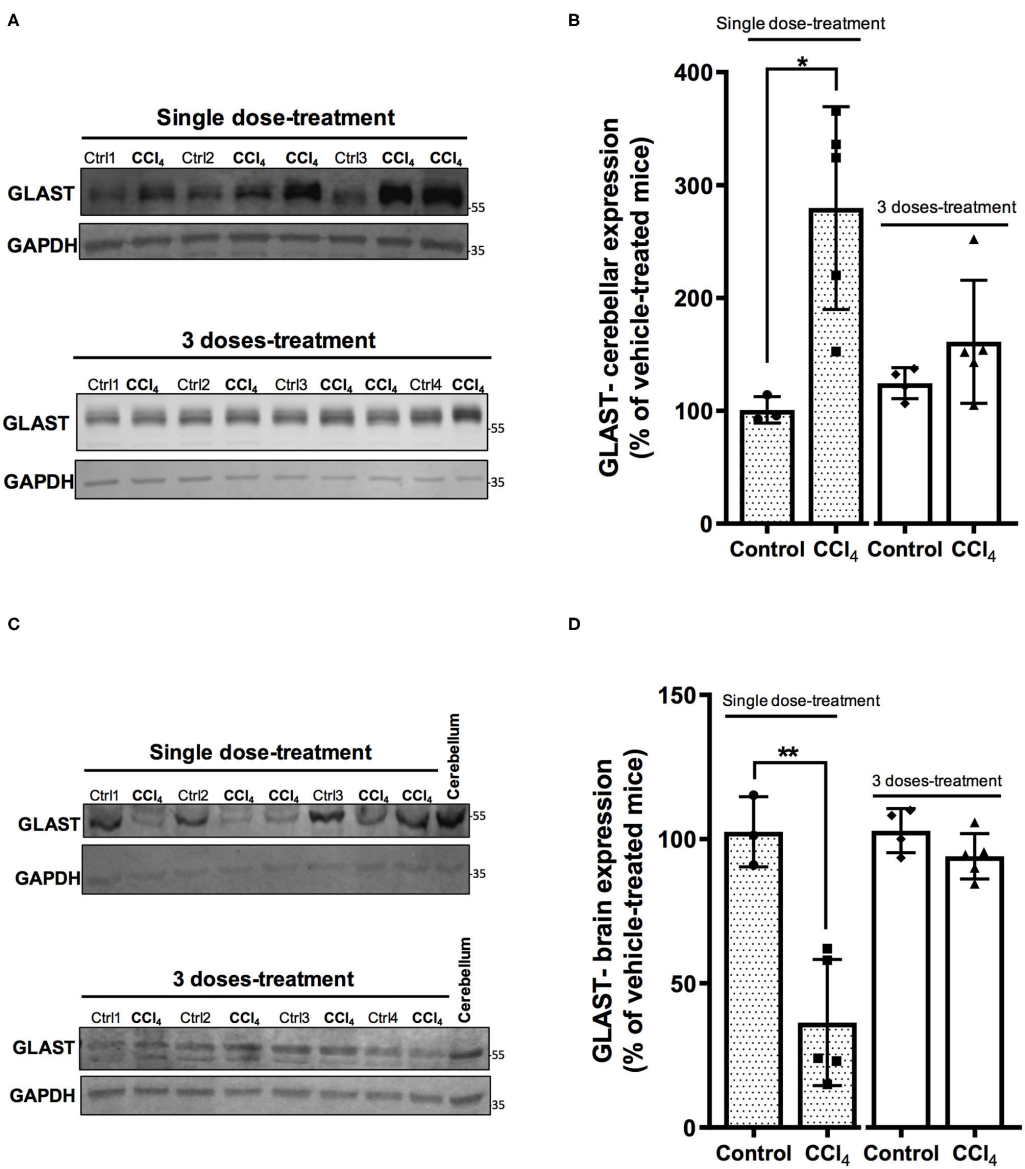
The expression pattern of GLAST/EAAT1 is altered in cerebellum **(A,B)** and brain **(C,D)** after CCl_4_ treatment. **(A)** GLAST/EAAT1 immunoreactivity in cerebellum from mice treated with one dose of CCl_4_ or three doses of CCl_4_. **(B)** Quantification of GLAST/EAAT1 protein expression signal intensity in cerebellum tissue. **(C)** GLAST/EAAT1 immunoreactivity in brain from mice treated with one dose of CCl_4_ or three doses of CCl_4_. **(D)** Quantification of GLAST/EAAT1 protein expression signal intensity in brain tissue. Results are expressed as mean ± SD (*n* = 3–5 animals per group). Cerebellum tissue from mouse was used as positive control for the detection of GLAST/EAAT1 expression. An unpaired *t*-test with Welch's correction was performed to compare between the groups, **p < 0.01, *p < 0.05 vs. vehicle-treated mice (control).

Glu as the major excitatory neurotransmitter has an important role in excitatory synaptic transmission (Martínez-Lozada and Ortega, 2015) and a deficit of its transport has been demonstrated in hepatic encephalopathy (Schmidt et al., 1990; Knecht et al., 1997; Norenberg et al., 1997). Accordingly, we did evaluate whether the expression of GLAST/EAAT1 and GLT-1/EAAT2, the main Glu transporters expressed in glial cells, is modulated in the CNS following liver injury. Protein levels of GLAST/EAAT1 transporter displayed a differential regulation depending on the brain region analyzed. Indeed, after a single dose of CCl_4_, protein expression of GLAST/EAAT1 was significantly increased in cerebellum by 270 ± 102.73% (*p* = 0.010) (Figures 4A,B), while a 79% diminished expression was observed in brain (Figures 4C,D). No significant differences in GLAST/EAAT1 were reported neither in brain or cerebellum after three doses of CCl_4_ (Figures 4A–D).

**Figure 5:**
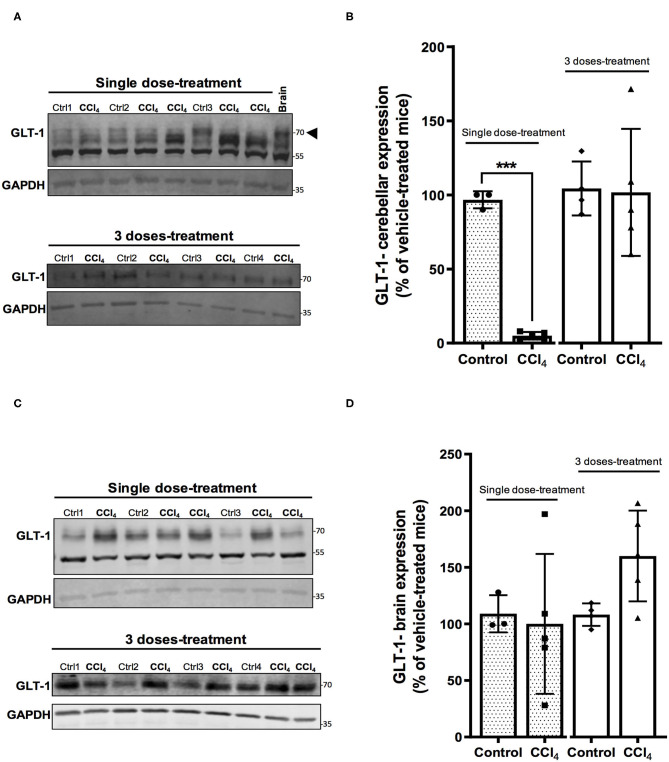
The GLT-1/EAAT2 protein expression is down-regulated in cerebellum after CCl_4_ treatment. **(A)** GLT-1 immunoreactivity in cerebellum from mice treated with one dose of CCl_4_ or three doses of CCl_4_. **(B)** Quantification of GLT-1/EAAT2 protein expression signal intensity in cerebellum tissue. **(C)** GLT-1/EAAT2 immunoreactivity in brain from mice treated with one dose of CCl_4_ or three doses of CCl_4_. **(D)** Quantification of GLT-1/EAAT2 protein expression signal intensity in brain tissue. Results are expressed as mean ± SD (*n* = 3–5 animals per group). Brain tissue from mouse was used as a positive control for the detection of GLT-1 expression. An unpaired *t*-test with Welch's correction was performed to compare between the groups, ***p < 0.0005 vs. vehicle-treated mice (control).

In parallel, GLT-1/EAAT2 protein expression was downregulated in cerebellum of mice treated with a single dose of CCl_4_ (Figures 5A,B), showing a reduction of 94% (*p* = 0.0004), while no effect was noticed in the group of mice treated with three doses of CCl_4_ (Figures 5A,B). Finally, no changes were detected in GLT-1/EAAT2 expression in brain tissue between vehicle- and CCl_4_ -treated mice (Figures 5C,D).

### Glutamine Synthetase Expression Is Modified in CNS After a Single Dose of CCl_4_

**Figure 6:**
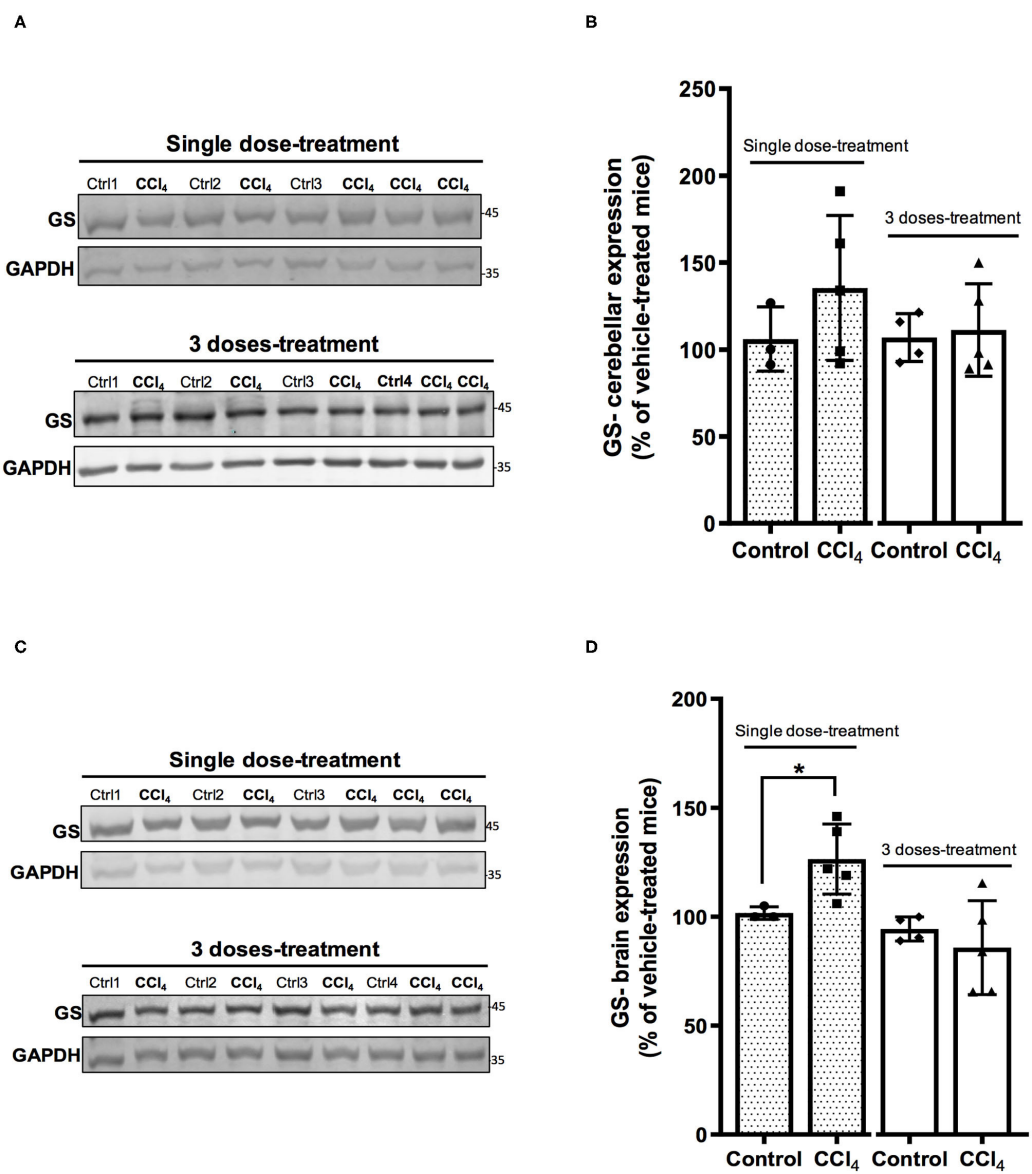
**One** dose of CCl_4_ increases GS protein expression in mouse brain. **(A)** GS immunoreactivity in cerebellum from mice treated with one dose or three doses of CCl_4_. **(B)** Quantification of GS protein expression signal intensity in cerebellum tissue. **(C)** GS immunoreactivity in brain from mice treated with one dose or three doses of CCl_4._ **(D)** Quantification of GS protein expression signal intensity in brain tissue. Results are expressed as mean ± SD (*n* = 3–5 animals per group). An unpaired *t*-test with Welch's correction was performed to compare between the groups, *p < 0.05 vs. vehicle-treated mice (control).

The analysis of expression of GS shows no changes in the protein levels in cerebellums from mice after the treatment with both doses of CCl_4_ as compared to vehicle-treated mice (Figures 6A,B). However, the protein levels of GS showed a slight increase in brain tissue in mice group treated with a single dose of CCl_4_ (122 ± 16.50%; *p* = 0.024) (Figures 6C,D) as compared to the mice group treated with the vehicle (101 ± 2.88%).

### Glutamate Transporters Expression Is Altered in Liver Tissue After Acute Injury

**Figure 7:**
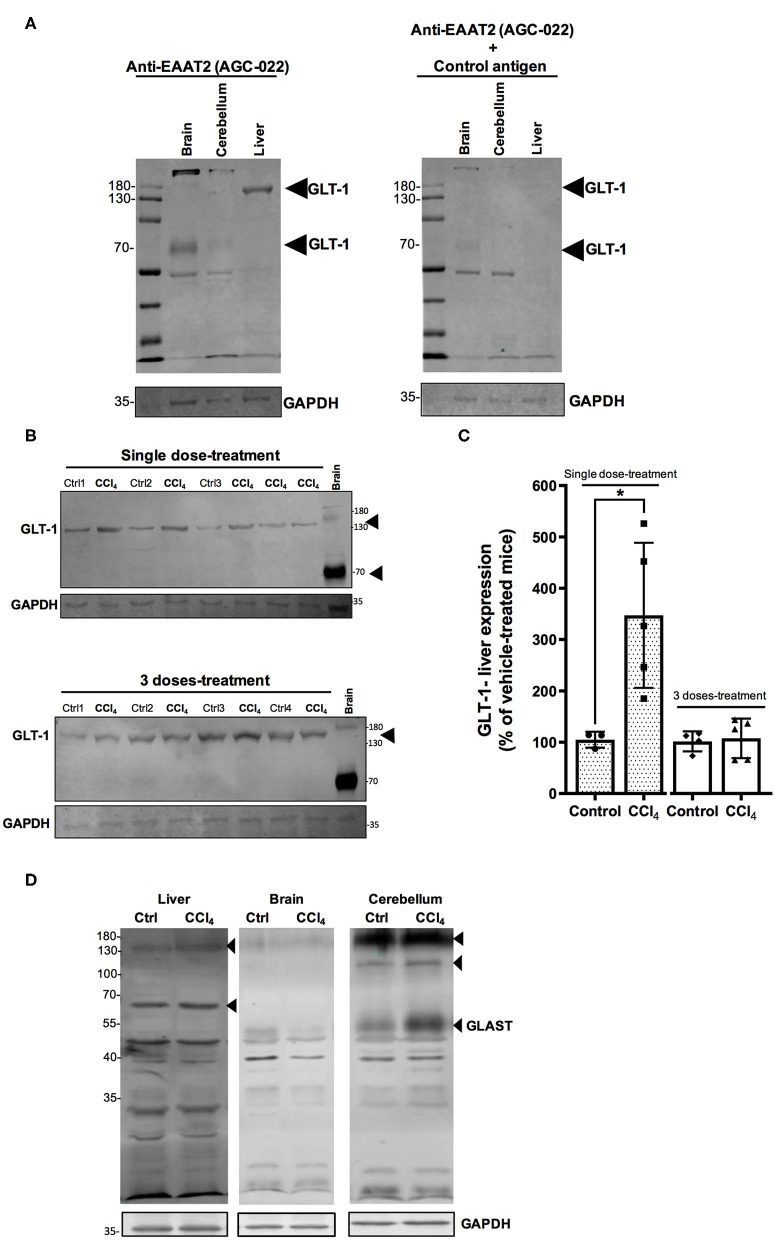
The protein levels of GLT-1/EAAT2 are upregulated in the liver after a single dose of CCl_4_ (1-day treatment). **(A)** Validation of GLT-1/EAAT2 protein expression in liver tissue homogenate. Immunoblotting was performed using Anti-EAAT2 (Table 1) and the control antigen provided by Alomone (AGC-022). Mouse brain and cerebellum tissues were used as positive controls for the detection of GLT-1/EAAT2. **(B)** GLT-1/EAAT2 immunoreactivity in liver from mice treated with one dose or three doses of CCl_4._ **(C)** Quantification of GLT-1/EAAT2 protein expression signal intensity in liver tissue. Mouse brain tissue was used as positive control. **(D)** Validation of GLAST/EAAT1 protein expression in liver tissue. Immunoblotting was performed using Anti-EAAT1 (Table 1). Mouse brain and cerebellum tissues were used as positive controls. Results are expressed as mean ± SD (*n* = 3–5 animals per group). An unpaired *t*-test with Welch's correction was performed to compare between the groups, *p < 0.05 vs. vehicle-treated mice (control).

GLT-1/EAAT2 expression has been reported in rat and mouse liver tissues (Berger and Hediger, 2006; Hu et al., 2018) and recently GLAST/EAAT1 was also reported in a human hepatoblastoma cell line, HepG2 (Jiménez-Torres et al., 2020). In this study, we validated the protein expression of Glu transporters, GLT-1/EAAT2 and GLAST/EAAT1, through an immunoblotting approach. When membranous protein fractions from brain and cerebellum tissues were separated *via* SDS-PAGE and immunoblotted with anti-EAAT2/GLT-1 antibody, several distinct bands (~70, 150, and 190 kDa) were detected in brain and cerebellum, while only a band corresponding to GLT-1/EAAT2 was detected at ~150 kDa in the liver tissue (Figure 7A). Its identity was confirmed when the blot of the protein extracts was pre-incubated with the control peptide (Figure 7A). The protein levels of GLT-1/EAAT2 were significantly upregulated 24 h after a single dose of CCl_4_ (434 ± 100.89%; *p* = 0.017) compared to vehicle-treated mice (104 ± 10.40%) (Figures 7B,C). This effect was lost in the mice group treated with three doses of CCl_4_, showing the percentage of protein expression as equivalent to the vehicle mice group (Figures 7B,C). In our experimental conditions, we were not able to detect any GLAST/EAAT1 immunoreactivity in the liver tissue (Figure 7D).

## Discussion

Acute hepatotoxicity of CCl_4_ is well-established in animal models (Scholten et al., 2015). Intraperitoneal administration of CCl_4_ induces elevated levels of fat, serum urea, liver enzyme activities, and histopathological evidence of liver damage with inflammation and cell necrosis mainly in the perivenular zone (Korsrud et al., 1972; Recknagel et al., 1989; Scholten et al., 2015). The data reported in Figure 2 and Supplementary Figure 1, confirmed the liver injury in the mice with one single and three doses of CCl_4_, by showing the histopathological disorganization in the intrahepatic parenchyma, the progression of collagen deposition as shown by Sirius Red staining, and the down-regulation of the protein expression of GS, exclusively expressed in perivenous hepatocytes and one of the two ammonia-detoxification systems aside from urea cycle in the hepatic acinus (Brosnan and Brosnan, 2009).

When an ALI happens in patients without a preexisting liver disease, it is associated with the development of neurologic disorders (Mans et al., 1994; Weissenborn et al., 2005; Ahboucha and Butterworth, 2008; Lee et al., 2008; Al-Bassam and Warrillow, 2018). Increased neurotoxin levels, impaired neurotransmission, changes in brain energy metabolism, systemic inflammatory response and alterations of the blood-brain-barrier (BBB), have all been suggested as key features in the pathophysiology of HE, a reversible syndrome of impaired brain functions that occur in patients with advanced liver damage (Ferenci, 2017). This suggests the potential role of these parameters in neurological pathology at the early stages of liver injury.

Glu, the main excitatory neurotransmitter, requires the involvement of both neurons and glial cells to elicit its function as a neurotransmitter, in what has been known as a tripartite synapse (Martínez-Lozada and Ortega, 2015). The levels of Glu in the synaptic cleft are tightly regulated mostly by glial EAATs (Danbolt, 2001), avoiding an overstimulation of Glu receptors and preventing an excitotoxic insult (Kew and Kemp, 2005). Glu taken up by astrocytes is converted to Gln by GS, released and internalized by neurons to be converted back to Glu in what is known as the Glu/Gln shuttle (McKenna et al., 2012; Martínez-Lozada and Ortega, 2015). Therefore, the balance in the components of this machinery is essential for an appropriate neurotransmission involving this amino acid. The major findings of this study show a differential Glu transporters expression in cerebellum in acute liver damage after a single CCl_4_ dose. While up-regulated for GLAST/EAAT1, the protein expression of GLT-1/EAAT2 is down-regulated. Such alteration in Glu transporters is accompanied by astrogliosis and Purkinje cells loss. Intriguingly, no changes were noticed after three doses of CCl_4_. Glu acts as a signaling molecule in glial cells and it is linked to transcriptional and translational control, critically involved in the control of Glu recycling and neurotransmission. Glial Glu transporters are key molecules that sense synaptic activity and by these means modify their physiology in the short and long-term (Martínez-Lozada and Ortega, 2015). In cerebral cortex, GLAST/EAAT1 protein expression is up-regulated in HE patients with cirrhosis (Görg et al., 2010). However, these effects have been poorly studied at cerebellar level. The glial Glu transporters GLAST/EAAT1 and GLT-1/EAAT2 are up-regulated in the molecular layer in the cerebellum of long-term portocaval-shunted rats (Suárez et al., 2000). It is well-known that GLAST/EAAT1 is the main transporter in cerebellum, most abundant in Bergmann glial cells in the cerebellar molecular layer and deep cerebellar nuclei (Rothstein et al., 1994). Therefore, even though we also reported here a down-regulation of GLT-1/EAAT2 expression in cerebellum and it is tempting to speculate, the upregulation of GLAST/EAAT1 protein levels detected after CCl_4_ acute exposure- as depicted in Figures 4A,B- may reflect an increase in the Glu uptake. By modulating the permanency of Glu in the extracellular space, this enhanced activity might promote a more efficient Glu recycling to maintain the neuronal communication in the first hours following the acute liver damage induced by CCl_4_. However, after three doses of CCl_4_, the effect on the expression of Glu transporters was not observed, suggesting that GLAST/EAAT1 expression is modulated at short-term might be through translational control (Martínez-Lozada and Ortega, 2015) consequently to the acute liver damage. These results are in sharp contrast to the reported GLAST/EAAT1 reduction in brain tissue in hyperammonemia and HE without liver disease, where a transitory down-regulation of GLAST, and increase of extracellular Glu is followed by an upregulation mechanism to counteract the excitotoxicity of Glu levels (Norenberg et al., 1997; Butterworth, 2002; Ochoa-Sanchez and Rose, 2018). Since the expression and activity of GLAST/EAAT1 in the prevention of an excitotoxic insult is related mainly to Purkinje cells survival, the damage found herein supports the scenario in which the neurotoxic cerebellar CCl_4_ effect is related to astrogliosis and Glu transporters disruption. Nevertheless, it is important to mention that Purkinje neuron loss could also result from CCl_4_-induced cytotoxicity since the loss of Purkinje cells was observed after both, single and three doses of CCl_4_. CCl_4_ is a lipid-soluble agent that is able to bind to lipids and proteins, suggesting that it might cross the BBB (Cohen, 1957). CCl_4_ intraperitoneal administration in mice has been shown to perturbate the expression of essential transmembrane proteins needed for the formation of tight junction strands such as claudin-5 which also promotes the BBB permeability (Baek et al., 2020). Furtheremore, it has been reported that brain tissue from an autopsy after CCl_4_ fatal intoxication revealed congestion of cerebral blood vessels associated with fatty degeneration, demyelination, Purkinje cell damage, and necrosis (Cohen, 1957; Luse et al., 1967). The earliest and severe aftermaths of CCl_4_ poisoning including headache, ataxia, vertigo, lethargy, coma (Luse et al., 1967), have been reported in some patients with ALI (Weissenborn et al., 2005; Al-Bassam and Warrillow, 2018). The mechanisms responsible for these neurological disorders in ALI at early stages are far from being fully understood; however, a multifactorial mechanism has been suggested, this includes direct effects of systemic and cerebral inflammation, recruitment of monocytes after microglial activation, release and brain accumulation of ammonia, lactate, and manganese, and altered permeability of the BBB (Butterworth, 2013). As in ALI, treatment with a single intraperitoneal injection of CCl_4_ at a lower dose (3.0 mL/kg of body weight) than the dose used in our study, increases TNF-alpha, IL-1b, and IL-18 levels in cerebral cortex, hippocampus, and cerebellum 4 h post-treatment (de Souza Machado et al., 2015), while CCl_4_ chronic treatment results in an upregulation of IL-1β and TGF-β1 mRNA levels in mouse cerebral cortex (Baek et al., 2020). Neuroinflammation induced in hyperammonemia has been associated with a disruption of neurotransmission in liver injury models by showing alterations in glutamatergic and GABAergic neurotransmission in the basal ganglia–thalamus–cortex circuit (Llansola et al., 2015), microglial activation and upregulation of the membranal expression of GABA transporter type 3 (GAT3). This augmentation is correlated to an increase in the cerebellar GABAergic tone leading to an impaired motor coordination (Hernandez-Rabaza et al., 2016). Hence, systemic and tissular factors might act synergistically in our model to induce the (~26%) loss of Purkinje cells. This loss might also reflect that the cerebellum is an important target in the early stages of acute liver damage induced by CCl_4_, since these effects are present after a single dose-treatment and could be associated with the altered motor activity and coordination observed in patients with HE. In line with this, a recent study has shown that the loss of Purkinje and granular neurons occurs in cerebellum at early stages of steatohepatitis (Balzano et al., 2018), contributing to the explanation of why neurological dysfunctions are still present in patients after liver transplantation (Campagna et al., 2014; Balzano et al., 2018).

Liver damage leading to disruption in the CNS has been previously demonstrated to be closely linked not only with microglial activation but also with upon reaction of glial cells as astrocytes, the central cells for Glu transport (Haroon et al., 2017). The data in this study also show an increase of GFAP protein levels in both cerebellum and brain tissue after a single dose of CCl_4_. In mouse primary astrocytes and microglia, NO induces GFAP expression (Brahmachari et al., 2006). Astrocytes express inducible NO synthase (iNOS) to produce an excessive amount of NO, a molecule closely linked to neuropathologies and neuroinflammatory conditions (Murphy, 2000). Since CCl_4_ induces high mRNA expression of iNOS and eNOS in rodents (Recknagel et al., 1989; Ji et al., 2018), it may be a plausible explanation for GFAP upregulation, however, it needs to be evaluated in future studies.

Another finding of this study is the up-regulated expression of GS in brain tissue noticed after a single dose of CCl_4_. In the brain, GS is primarily located in astrocytes and carries out the amidation of Glu, achieving ammonia detoxification (Bessman, 1964). Glu has been reported to induce an increase of GS activity as shown in cultured rat astrocytes (Wu et al., 1988; Tiffany-Castiglioni et al., 1989) and mRNA levels as demonstrated in co-cultures of rat cortical astrocytes with cerebellar granular cell neurons (Mearow et al., 1990). In this study, a reduction of GLAST/EAAT1 expression was found, while no apparent change in protein levels of GLT-1/EAAT2 was detected in brain tissue. GLT-1/EAAT2 is the main transporter in brain cortex (Rothstein et al., 1994), therefore this may suggest that Glu influx is not impaired in the forebrain region at the early stages of liver injury by CCl_4_. It has been demonstrated that the activity of GS is also regulated by ammonia in time and concentration-dependent manner in cortical astrocytes cultures, without alterations in the removal of Glu from the extracellular space, enhancing the metabolism of accumulated Glu and stimulating the conversion of Glu to Gln (Waniewski, 1992) to protect against neuronal degeneration.

Our results support a plausible glutamatergic disruption by the modification of the expression of key proteins in the Glu/Gln shuttle in CNS consequently to acute liver damage. Furthermore, *in vivo* and *in vitro* studies have demonstrated the disruption of Glu transport also in the liver, both in models of liver diseases (Najimi et al., 2014) and after xenobiotic exposure (Jiménez-Torres et al., 2020). Is the Glu uptake disrupted in the liver at the early stages of the liver injury? To shed some light on this issue, we analyzed the protein expression of both GLAST/EAAT1 and GLT-1/EAAT2 in liver tissue after CCl_4_ treatment. The results described above revealed that as previously reported, GLT-1/EAAT2 is the main Glu transporter expressed in mouse liver (Utsunomiya-Tate et al., 1997; Berger and Hediger, 2006; Hu et al., 2018). The detection of GLT-1/ EAAT2 as several bands in CNS (~70, 150, and 190 kDa) and only one band in liver tissue (~150 kDa) indicates that this higher molecular weight band might be corresponding to a multimeric fraction of the transporter. Previous studies have indicated that GLT-1/EAAT2 exists as homomultimer, whereas GLAST/EAAT1, may also exist as dimers (Haugeto et al., 1996). Although the mechanism and the effects of these clustering are not been well-understood, it has been suggested that, in addition to GLT-1/EAAT2 clustering, other signaling molecules or scaffolding proteins may be recruited to this cluster forming multimeric complexes, and it may promote the decrease in both cell surface expression and activity of GLT-1/EAAT2 (Zhou and Sutherland, 2004). Our results demonstrated that the expression of GLT-1/ EAAT2 might be modulated upon ALI; in sharp contrast to the CNS, the role of Glu in the liver is mainly metabolic due to its use as a substrate in several metabolic pathways. GS and GLT-1/EAAT2 transporter are co-localized in the perivenous hepatocytes (Kuo et al., 1991; Brosnan and Brosnan, 2009). However, in contrast to our findings on down-regulation of GS protein levels, the up-regulation of GLT-1/EAAT2 after a single dose of CCl_4_ might be involved in restoring the intracellular pools of Glu in the liver for cellular homeostasis as an early response to acute damage.

## Conclusion

Taken together, our results demonstrate a disruption of Glu transporters in the liver-brain axis at the early stages (24 h) of acute liver injury. ALI induced by CCl_4_ is associated with a neuronal cell loss, glial activation, and imbalance in the expression of key proteins in the Glu recycling, such as Glu transporters, and GS mainly in cerebellum. The expression of the main Glu transporter in liver mouse tissue, GLT1/EAAT2, is also up-regulated upon liver toxicity. These effects, observed only in the first hours following the CCl_4_-induced damage with a single dose but not in the group treated with three doses of CCl_4_, suggest a short-term regulation of Glu transporters in the CNS. Further studies, currently in progress in our laboratories are aiming to determine whether the changes in the protein expression of these molecules reflect a modification in the Glu uptake activity and the activity of GS which is closely linked with ammonia detoxification to decipher the mediators of this inter-organ crosstalk.

## Data Availability Statement

The raw data supporting the conclusions of this article will be made available by the authors, without undue reservation.

## Ethics Statement

The animal study was reviewed and approved by Local ethical review board of the Institut de Recherche Expérimentale et Clinique (IREC), UCLouvain (2015/UCL/MD/12).

## Author Contributions

CJ-T performed the majority of experiments, analyzed the data, and wrote the first draft paper. HE-K and LH-K participated in performing CCl_4_ treatments, recovery tissue samples from mice, and immunohistochemistry experiments. ES, AO, and MN were involved in revising the manuscript. AO and MN coordinated the research. MN designed the research and wrote the final version of the manuscript. ES and AO provided financial support. All authors contributed to the article and approved the submitted version.

## Conflict of Interest

The authors declare that the research was conducted in the absence of any commercial or financial relationships that could be construed as a potential conflict of interest.

## Supplementary Material

Supplementary Figure 1 Validation of hepatocellular injury after CCl_4_ exposure. **(A)** Hematoxylin-eosin staining in periportal and perivenous areas of liver sections from mice treated with the vehicle, one dose or three doses of CCl_4_. Liver sections from mouse treated with the vehicle show normal hepatocytes architecture. Liver sections of mouse treated with one dose of CCl_4_ show necrosis in centro-lobular area (black arrows). Note the discrete inflammatory foci, composed predominantly of lymphocytes (white arrows). Liver sections from mouse treated with three doses of CCl_4_ show dead hepatocytes and inflammatory foci around centro-lobular area (white arrows). **(B)** Picro Sirius Red staining in liver tissue from mice treated with the vehicle, one dose or three doses of CCl_4_. Liver sections from control mouse treated with the vehicle show very collagen fibers detection. Liver section from mouse treated with a single dose of CCl_4_ showing a slight increase in collagen deposition around the centro-lobular area. Liver section of treated mouse with three doses of CCl_4_ show an excessive collagen deposition around the centro-lobular area. Representative images of 3–5 animals per group were shown. Scale: 50 μm.

## Abbreviations

CCl_4_: Carbon tetrachloride
ALI: Acute liver injury
HE: Hepatic encephalopathy
Glu: Glutamate
NMDA: N-methyl-D-aspartate
AMPA: Alpha-amino-3-hydroxy-5-methyl-4 isoxazolepropionic acid
EAAT: Excitatory Amino Acid Transporter
GS: Glutamine synthetase
Gln: Glutamine
CNS: Central nervous system
BBB: Blood brain-barrier
NO: Nitric oxide
H&E: Hematoxylin and eosin
α-SMA: α –Smooth muscle actin
GFAP: Glial fibrillary acid protein
GAPDH: Glyceraldehyde-3-phosphate dehydrogenase
GLAST: Glutamate aspartate transporter
GLT-1: Glutamate transporter 1
GAT3: GABA transporter type 3.
