# Supplementary figures and images for: Acute Liver Toxicity Modifies Protein Expression of Glutamate Transporters in Liver and Cerebellar Tissue
Source: Front Neurosci. 2021 Jan 6;14:613225. doi: 10.3389/fnins.2020.613225 (PMC7815688; doi:10.3389/fnins.2020.613225)

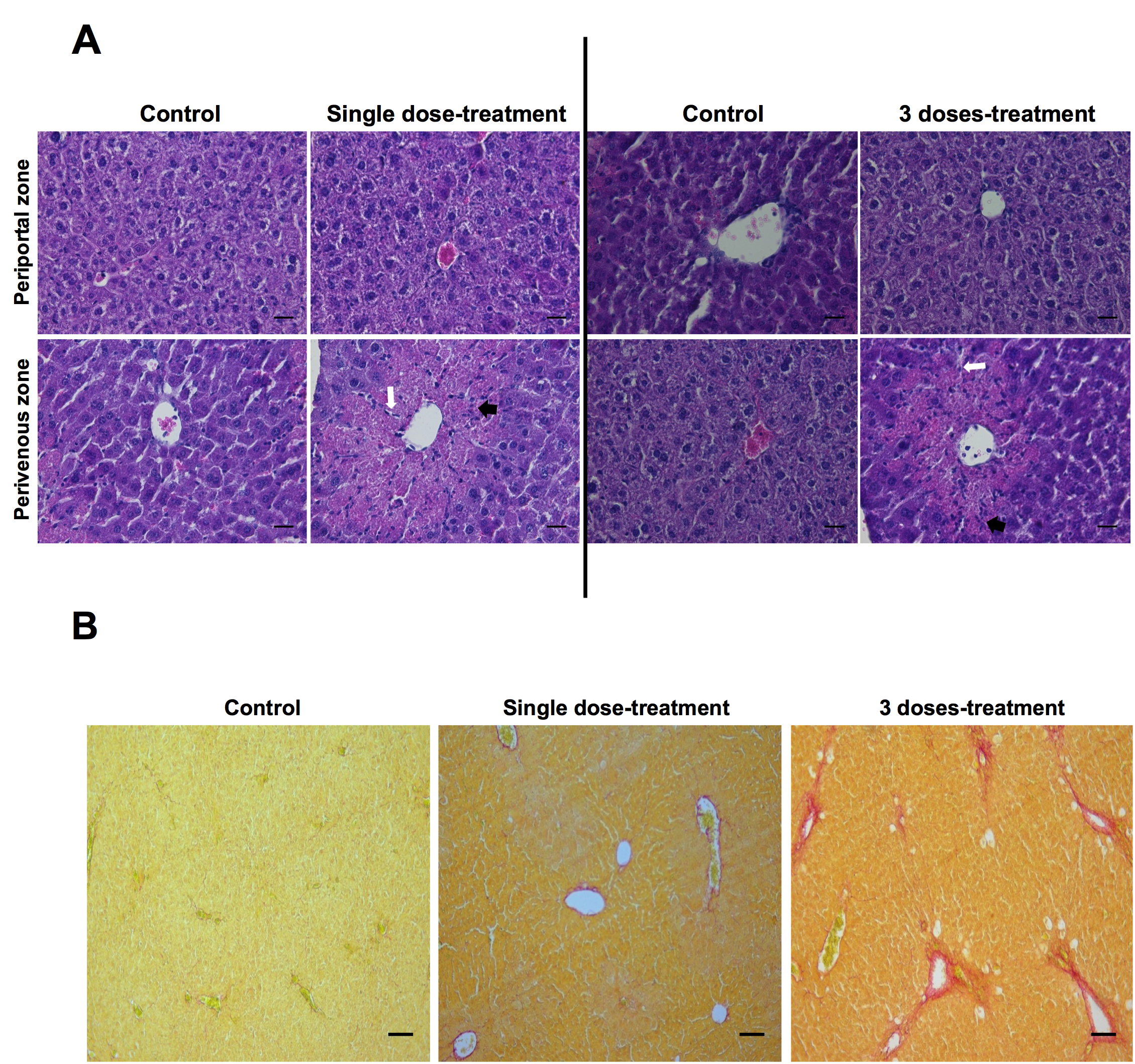

Supplement: Supplementary Figure 1 — Validation of hepatocellular injury after CCl4 exposure. (A) Hematoxylin-eosin staining in periportal and perivenous areas of liver sections from mice treated with the vehicle, one dose or three doses of CCl4. Liver sections from mouse treated with the vehicle show normal hepatocytes architecture. Liver sections of mouse treated with one dose of CCl4 show necrosis in centro-lobular area (black arrows). Note the discrete inflammatory foci, composed predominantly of lymphocytes (white arrows). Liver sections from mouse treated with three doses of CCl4 show dead hepatocytes and inflammatory foci around centro-lobular area (white arrows). (B) Picro Sirius Red staining in liver tissue from mice treated with the vehicle, one dose or three doses of CCl4. Liver sections from control mouse treated with the vehicle show very collagen fibers detection. Liver section from mouse treated with a single dose of CCl4 showing a slight increase in collagen deposition around the centro-lobular area. Liver section of treated mouse with three doses of CCl4 show an excessive collagen deposition around the centro-lobular area. Representative images of 3–5 animals per group were shown. Scale: 50 μm. [file Image_1.TIFF]
